# Application of Platelet-Rich Fibrin as Regeneration Assistant in Immediate Auototransplantation of Third Molar with Unformed Roots: Case Report and Review of Literature

**DOI:** 10.1155/2020/8170646

**Published:** 2020-01-21

**Authors:** Hamzah Alkofahi, Alaa Maghaireh, Mamoon Fnaish, Mohammad Jarrah, Mohammad Bataineh

**Affiliations:** ^1^Department of Oral and Maxillofacial Surgery, Jordanian Royal Medical Services, Irbid, Jordan; ^2^Department of Periodontics, Jordanian Royal Medical Services, Irbid, Jordan; ^3^Department of Endodontics, Jordanian Royal Medical Services, Irbid, Jordan

## Abstract

**Background:**

Autogenous Tooth Transplantation (ATT) is the surgical movement of a maturely or immaturely formed tooth from its original site to another extraction site or a surgically prepared socket in the same individual. The most important factor in the healing process after autotransplantation is the presence of intact and viable periodontal ligament cells, which have the ability to differentiate into osteoblasts and able to induce bone production. ATT can successfully replace removable dentures as a restoration option in a growing patient, while implants can be placed only after skeletal maturity is attained. *Case Presentation.* In this case, we presented an immediate ATT of the third molar with unformed roots to the extraction socket of the first molar with evidence of continued root formation after 2 years of follow-up.

**Conclusion:**

Platelet-Rich Fibrin (PRF) can induce sustainable and accelerated healing, and it can also induce the regeneration process of the periodontal tissues and pulpal formation. This process plays a key role in future root development and success rate.

## 1. Background

Autogenous Tooth Transplantation (ATT) is the surgical movement of a maturely or immaturely formed tooth from its original site to another extraction site or a surgically prepared socket in the same individual [1–3]. Vidman et al. in 1915 was the first to report autotransplantation [4]. Since then, the procedure has gained popularity in premolars and canines. Slagsvold and Bjercke in 1974 reported the results of the autotransplantation of 34 premolars with incompletely formed roots done in the period between 1959 and 1970 with a mean follow-up duration of 6 years. He showed a 100% survival rate with the maintained ability of the transplanted teeth to complete root development [5].

The biology of successful autotransplantation depends on the ability of root periodontal (PDL) cells to differentiate and induce dentine and cementum formation. PDL cells have the ability to differentiate into osteoblasts and are able to induce bone formation. Andreasen et al. showed that the presence of intact and viable periodontal ligament cells is considered to be the most important factor to have a successful healing process after autotransplantation [6]. PDL healing has a better prognosis in the case of incomplete roots transplanted in fresh extraction sockets than in the case of surgically formed sockets [7–9].

Many reports have emphasized that the optimum time to achieve a successful ATT is when the tooth root has reached two-thirds to three-quarters of its expected length. However, other factors play an important role including (1) performing atraumatic extraction to preserve Hertwig's root sheath and insure future root growth [10], (2) decreasing the duration of the teeth out of socket before implantation [11], (3) having the apical foramen dimensions > 1 mm to increase the probability of postoperative revascularization [12], and (4) maintaining a good alveolar bone support at the time of ATT [13].

ATT can be a permanent aesthetic restoration for patients who are not suitable for dental implants or fixed prosthesis. It has the advantage of low cost and the ability to move the teeth orthodontically in the future, if needed. Moreover, patients who undergo ATT maintain normal chewing and arch integrity [14], pulpal viability, and preserve periodontal ligament health, maintain normal proprioception reflexes, and stimulate eruption in growing patients [15]. ATT has the ability to preserve bone level and induce alveolar bone formation, which is important to keep the dental implant viable after the patient completes growth, even in the case of failure [7, 13, 16].

There are other types of autotransplantation including intra-alveolar transplantation, i.e., when the position of the tooth is changed within the original socket, e.g., surgical uprighting and intentional replantation, and when the tooth is replanted in the original socket after intentional extraction for treatment of endodontic lesions [17]. ATT is classified based on the time of procedure after the extraction of the recipient tooth, i.e., immediate or delayed transplantation after an initial phase of healing [18].

ATT is indicated to replace missing congenital teeth. In most of these cases, the source of the donor tooth is the crowding in the opposing arch [16]. ATT of lower premolars transplanted in the upper maxillary incisor sockets after traumatic tooth loss were also reported successfully [19]. ATT can be used for the reconstruction of marginal mandibular resection assisted with orthodontic treatment. Osterne et al. reported a successful case of the reconstruction of a mandibular alveolar bone defect in the region of the lower left canine and premolars post ameloblastoma resection with autotransplantation of the immature third molars followed by orthodontic treatment [11].

There is a high incidence of first molar loss in the pubertal patient because of caries and periodontal problems [7, 14, 20]. In these cases, ATT can successfully replace removable dentures, while implants can be placed only after skeletal maturity is attained [21].

In this case report, we present an immediate ATT of the third molar with unformed roots in the extraction socket of a first molar. We used Platelet-Rich Fibrin (PRF) to accelerate the healing and regeneration process of the periodontal tissues and pulpal formation. This process plays key role in future root development and success rate.

### 1.1. Case Report

**Figure 1:**
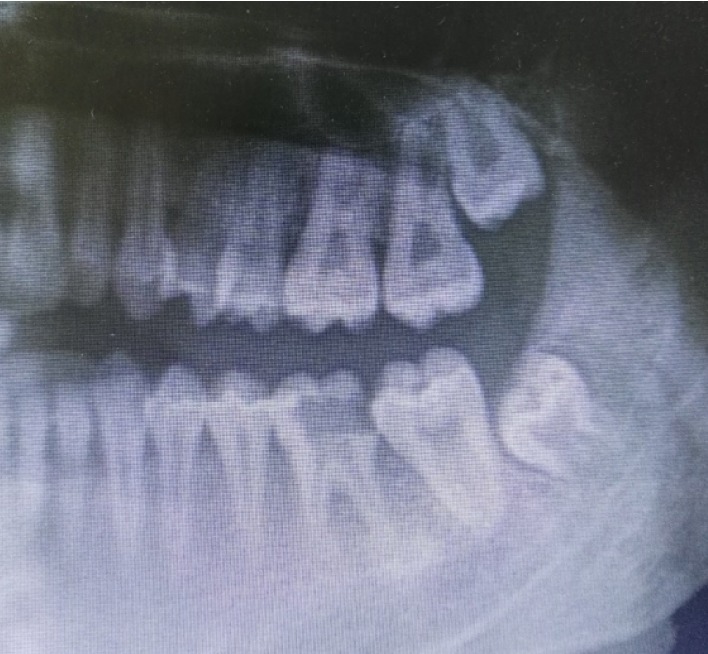
Recipient tooth number 19 with deep caries and donor tooth number 16 with unformed roots.

A 16-year-old female patient presented in the Oral and Maxillofacial Surgery Clinic at the Royal Medical Services of Jordan-Prince Rashid Hospital. The patient was referred from the Conservative Clinic at the same institution complaining from pain and tenderness on percussion at the area of tooth number 19. The patient's medical records showed no medical history. Dental examination has showed that the patient has extensive class II caries into subgingival level in tooth number 19 with necrotic pulp. Panoramic radiograph showed a periapical condensing osteitis with deep class II caries of tooth number 19 as shown in Figure 1. After consulting with the endodontist, several treatment options were presented including root canal treatment with crown lengthening, extraction followed by ATT, or extraction followed by future implant or fixed prosthesis. The patient and her parent were interested in ATT, after considering the low cost in comparison to the implant option. Hence, the decision was made to extract tooth number 19, followed by ATT and application of PRF at time of transplant.

### 1.2. Treatment

A consent form authorizing the procedure and explaining treatment risks and complications was signed by the patient's parent. On the day of ATT and one hour before the extraction, PRF was prepared according to Choukroun's protocol. Ten cc of venous blood was withdrawn, and blood was centrifuged at 3000 rpm for 10 minutes. The sealed tube was stored at 4°C and ready to use.

### 1.3. Extraction of Tooth Number 19

**Figure 2:**
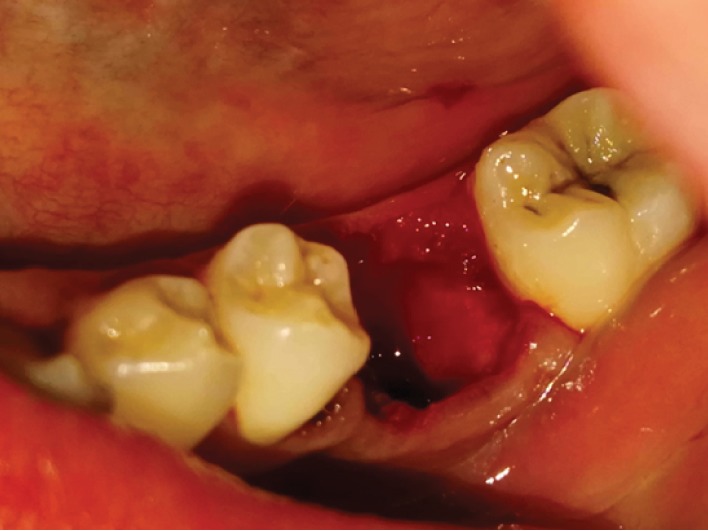
Recipient tooth number 19 with deep caries and donor tooth number 16 with unformed roots.

The patient underwent local anesthesia of the left inferior alveolar, lingual, and buccal nerves with 2 ampules of 1.8 mm of 2% articaine with 1 : 100,000 epinephrine. Once a profound anesthesia was confirmed, atraumatic extraction of tooth number 19 was done by straight elevator with minimal luxation. Extraction socket walls and apexes were cleaned from any granulation tissue by curettage of the socket and copious irrigation with 0.9% saline (Figure 2).

### 1.4. Extraction of Tooth Number 16

The decision to choose tooth number 16 as donator tooth was done following a radiographic assessment of root development and crown width mesiodistally of all third molars compared to the crown width of tooth number 19. The patient underwent local anesthesia of tooth number 16 through local infiltration of the posterior superior alveolar nerve and the greater palatine nerve with 2 ampules of 2% articaine with 1 : 100,000 epinephrine.

After confirming profound anesthesia, tooth number 16 was exposed using a small mucoperiosteal flap with mesial releasing incision distal to tooth number 15 with minimal bone removal. Tooth number 16 was moved with caution to the attached follicle and was stored in 0.9% saline. Copious irrigation of the transplant socket with 0.9% saline and smooth bony edges were confirmed before closure of the flap with interrupted 4/0 vicryl sutures.

### 1.5. ATT of Third Molar in Tooth Number 19 Socket

**Figure 3:**
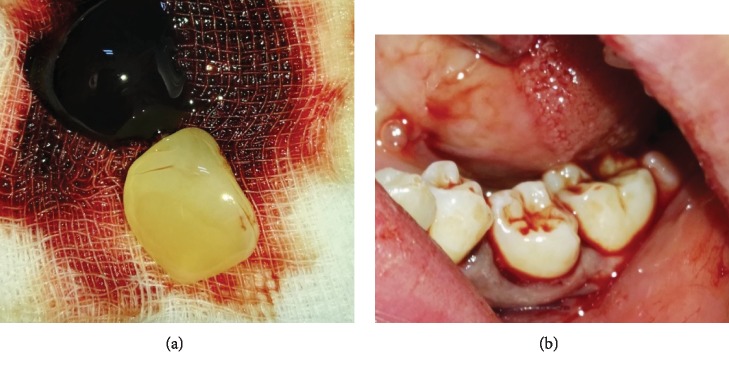
(a) PRF ready for application. (b) Donor third molar at place ready for splinting.

**Figure 4:**
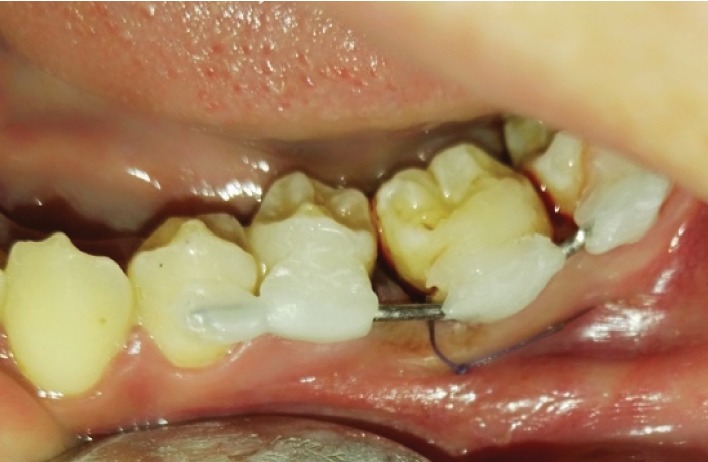
Rigid splinting of third molar.

At the transplanted socket, PRF was separated from red corpuscles and placed in the socket. The donator third molar was positioned on the recipient site and adjusted to 1 mm infraocclusion position by trimming interradicular bone, then returning back to saline. The implanted tooth was secured with 0.7 mm wire and composite resin from tooth number 18 to number 20 (Figures 3 and 4).

**Figure 5:**
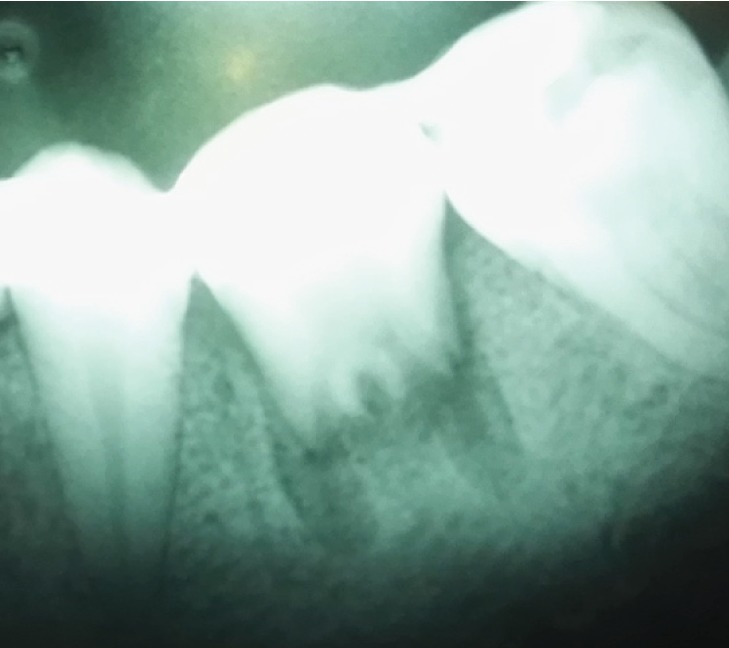
Immediate postoperative radiograph showing third molar fitting well in tooth number 19 socket.

Immediate postoperative periapical radiograph was taken to ensure the accurate position of the transplanted tooth in the recipient site as shown in Figure 5. In addition to postextraction instructions, the patient was instructed to rinse with 0.12% chlorhexidine gluconate mouthwash two times/day and was prescribed acetaminophen 500 mg tablets three times/day for 3 days, then PRN.

### 1.6. Postoperative Follow-Up

During the follow-up visit on day 7 postoperatively, the healing of the donor site and the implanted tooth were examined and no issues were reported. Subsequent follow-up was done on a weekly basis. During the 4-week follow-up visit, the splint was changed from nonrigid to flexible orthodontic wire. Subsequent follow-up visits were done at 3, 6, and 12 months.

**Figure 6:**
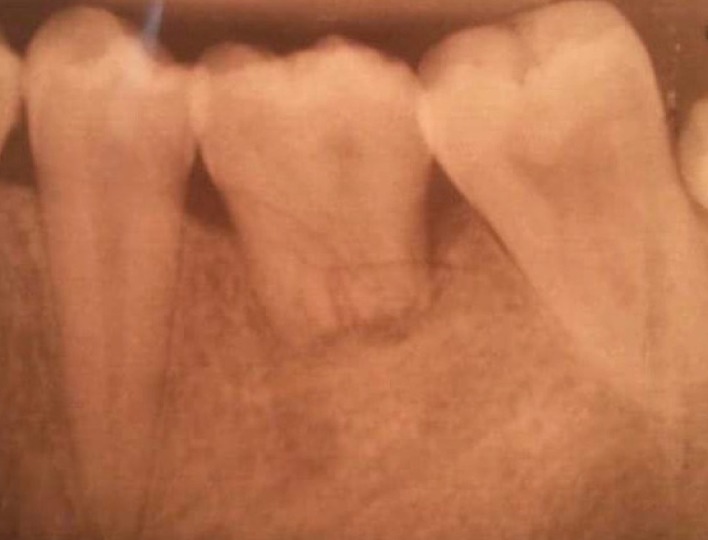
Periapical radiograph showing root formation 1 year after ATT.

**Figure 7:**
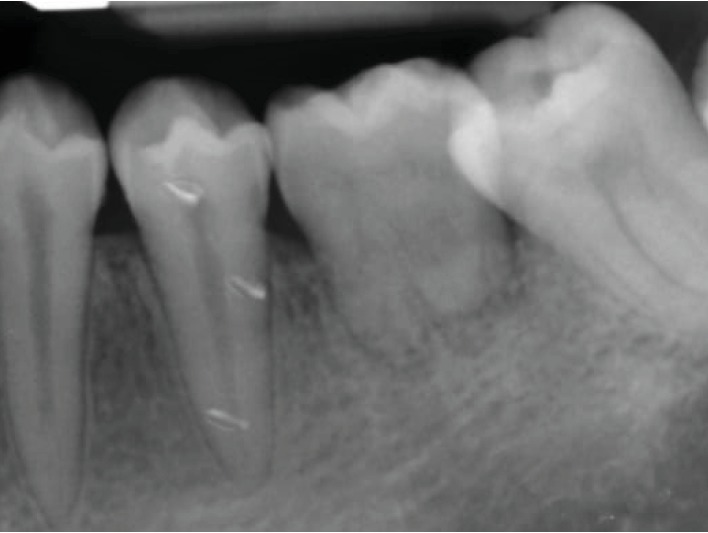
2-year periapical radiograph showing increased root length and open apex.

**Figure 8:**
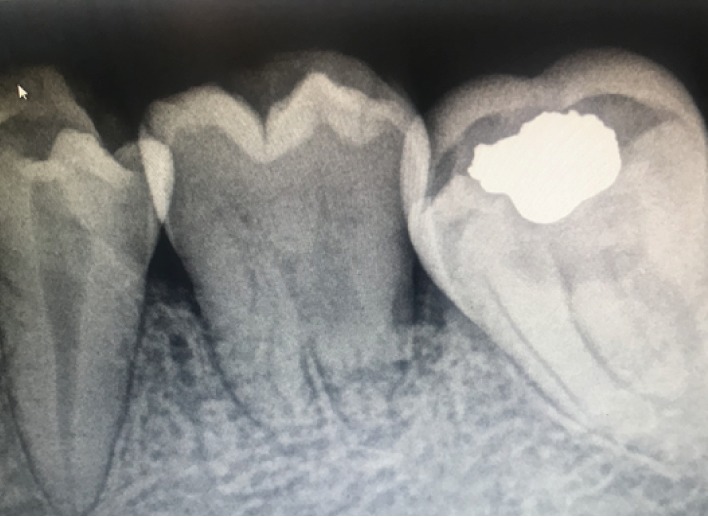
3-year periapical follow-up radiograph.

One year later, the implanted tooth showed continuous root formation as shown in Figure 6, with normal periodontium, tooth mobility, and vital pulp testing. The patient was satisfied and did not report any complaint. On the 2-year follow-up visit, periapical radiograph showed continuous root development reaching >1 root/crown ratio with open apex (Figure 7), and no signs of root resorption confirming the previous findings (Figure 8).

## 2. Discussion

Tooth loss during puberty age especially the first molar is not uncommon. In such cases, limited treatment options are available, such as ATT and implants. Implants are not indicated because they lack the ability to grow and may result in unsightly infraocclusion if placed during growth spurt. Such growth issues warrant the consideration of refined treatment choices with growth-friendly treatment modalities in growing children and/or adolescents.

ATT is considered as a reliable treatment option with proven success. However, for reporting the long-term evaluation of results, the researchers use the survival rate for describing the percentage of transplanted teeth still present at the time of examination [13]. On other hand, successful ATT should fulfil specific criteria including viable tooth with stable occlusion, no pathological mobility or pocket probing depths, and without evidence of ankylosis, root resorption, or infection [22, 23].

Schwartz et al. in 1985 [24] reported the survival rate of 210 autotransplanted teeth and found a 76.2% survival rate following 5 years postoperatively, while the 10-year survival rates dropped to 59.6%. A more recent study by Andreasen et al. [12], evaluating the long-term survival rate and pulpal healing of 370 autotransplanted premolars, with complete and incomplete root formation, has showed that survival rates of the completed root teeth is 98% and 95% for the uncompleted root teeth after an observation period ranging from 1 to 13 years. Andreasen et al. noticed that pulpal healing was highly related to the stage of root development at the time of transplantation. Teeth transplanted with incomplete and complete root formation showed 96% and 15% pulp healing, respectively.

Other studies showed that the ATT success rate may vary between mature and immature teeth. Kugelberg et al. [25] reported a 96% success rate for 23 immature teeth and 82% for 22 mature teeth following 4 years of follow-up. In 2010, Yan et al. [26] and Mensink and Van Merkesteyn [27] reported a 100% success rate of open apex teeth following more than 4 years.

The most recent systemic review and meta-analysis by Rohof et al. [28] in 2018 showed that ATT is a reliable treatment option with survival and success rates of autotransplantation of immature teeth >95% and with complications rates <5% in terms of ankylosis (2.0%), root resorption (2.9%), and pulp necrosis (3.3%).

Meta-analyses, evaluating outcomes of autotransplanted teeth with complete root formation, showed 2% annual failure rate, 2.1% root resorption rate, and 1.2% ankyloses rate with 1- and 5-year survival rates of 98% and 90.5%, respectively [28, 29].

Although ATT of mature and immature teeth is associated with a high success rate, endodontic treatment is usually necessary in mature teeth within 4 weeks to prevent development of pulp-associated lesion. On the other hand, ATT of immature teeth showed the ability of continuous pulp healing and reinnervation. Andreason et al. found that pulpal healing was the usual finding in teeth with immature root formation. He found necrotic pulp that necessitated endodontic treatment in all autotransplanted teeth with completely formed roots. However, according to Denys et al., the teeth with a root length less than half is associated with increased risk for arrested root development rather than more mature ones. His recommendation is that two-thirds to three-quarters of the expected root length is needed to optimize outcomes [30].

PRF was introduced by Choukroun et al. in 2001 as the second generation of platelet concentrates, composed of autologous leukocyte-platelet-rich fibrin matrix and containing various mitogenic factors such as platelet-derived growth factor, vascular endothelial growth factor, and transforming growth factor released by an *α* granule [31]. According to Choukroun, PRF is prepared simply by centrifuging a patient's own blood at 3000 rpm for 10 minutes without the need for thrombin or anticoagulant additives [31]. After centrifuging, PRF collected as the middle layer containing growth factors [32]. PRF stimulates angiogenesis through migration, division, and phenotypic change of endothelial cells. It also promotes cell mitosis and induces osteogenesis without inflammatory reactions. These effects act in a slow sustained process for at least one week [33] and up to 4 weeks [34, 35]. Dohan Ehrenfest et al. showed that PRF can induce a strong and continuous differentiation and stimulation of osteoblasts for 14 days with fibroblasts [36].

Moreover, PRF has shown successful results when used as a sole agent in periodontal regeneration like clinical attachment loss and intrabony defects [37, 38]. PRF has also shown to be effective in regenerative endodontics. Wang et al. evaluated the effect of PRF in the regenerative therapy of immature permanent canines [39]. PRF was able to increase the thickness of dental-associated mineral tissue. After 12 months follow-up, Bakhtiar et al. reported radiographic evidence of further root development and apical closure in 4 immature teeth with necrotic pulps [40].

Root formation and development are induced by Hertwig's epithelial root sheath (HERS), which is a bilayered epithelium that functions as a stimulator of mesenchymal cell differentiation into odontoblasts and cementoblasts [41]. Within the scope of PRF effects in dental regeneration, the possible mechanism for root development after transplanting third molars with less than a quarter root formed could be explained by the fact that PRF contains a dense fibrin network and a concentration of many growth factors like platelet-derived growth factor and vascular endothelial growth factor in sustainable way. The most important factor is transforming growth factor *β*1 (TGF*β*1), which is also secreted by HERS that induces positively the differentiation of dental papilla cells into odontoblasts ensuring a suitable environment for PDL cells to proliferate and the synthesis of an extracellular matrix [42].

Application of PRF in ATT has been reported successfully in 2 case reports followed for 6 months. Robindro Singh et al. has used PRF in the ATT of an impacted central incisor in a prepared socket as the second stage after the excision of odontoma. He applied the PRF membrane around the tooth at the time of ATT. His 6-month follow-up showed successful results with signs of root developments [42]. Devi et al. reported the immediate ATT of an immature third molar in the socket of an adjacent tooth. Radiographic and clinical examinations have shown successful results [3]. In addition to normal mobility and pocketing examination, two years of radiographic evaluation showed continued root development, as shown in the 3-year follow-up periapical radiograph after transplantation with other favorable clinical measurements.

## 3. Conclusion

From the presented case, we conclude that the benefits of including PRF in autotransplantation of teeth with immature roots, even when less than a quarter of the roots have formed, acts positively during the immediate and late regeneration process. PRF can eliminate the risk of arrested root formation and the need for pulpal treatment and can decrease the risk of complications.
